# Analysis of the effects of pesticides and nanopesticides on the environment

**DOI:** 10.1186/1753-6561-8-S4-P100

**Published:** 2014-10-01

**Authors:** Caroline Nishisaka, Renato Grillo, Gabriela Sanches, Leonardo Fraceto, Renata Lima

**Affiliations:** 1 Biotechnology, University of Sorocaba, Sorocaba, SP, Brazil; 2 Graduate in Functional and Molecular Biology, State University of Campinas, Campinas, SP, Brazil; 3 Bioprocess and Biotechnology Engineering, University of Sorocaba, Sorocaba, SP, Brazil; 4 State University of São Paulo, Sorocaba, SP, Brazil

## 

One of the main consequences of population growth is that an equivalent food production increase is needed. To meet this basic need of society and ensure growth in food production, it is necessary the use of agrochemicals such as herbicides, preventing competition between crops and weeds for soil nutrients. This growth in use of agrochemicals has historically had undesirable consequences due to their indiscriminate and sometimes reckless use, with health problems for farmers and environmental damage. One of the possible solutions to increase agricultural production without these consequences would be the application of nanotechnology to allow a safer use of pesticides and lessen health and environmental side-effects. However, the use of nanotechnology demands an investigation of possible toxic effects of this technology, mainly in relation to contamination of soil and water. The study we performed aimed to produce nanoparticles containing the herbicide paraquat and to analyze its possible genotoxic effects. The technique used was cytogenetic test of *Allium cepa *treated with nanoparaquat, conventional paraquat, tripolyphosphate chitosan nanoparticles, which were made in duplicate with and without humic substances. All concentrations were 0,38 mg.mL^-1^, and negative control was made with ultrapure water and humic substances for comparative purposes. Initial results indicated less chromosome damage in nanoparaquat treated samples compared to conventional paraquat herbicide, indicating that nanoencapsulation is a viable option as an attempt to minimize damage caused by paraquat.

## References

[B1] Larini L Manole Ltda Toxicologia dos Praguicidas In Toxicologia dos Praguicidas 1999 1 1 São Paulo: Manole Ltda 136 137

[B2] Fiskesjö G The Allium test as a standard in environmental monitoring Hereditas 1985 102 1 99 112 3988545 10.1111/j.1601-5223.1985.tb00471.x

[B3] Rocha JC Rosa AH Rocha JC Importância das Substâncias Húmicas no Ambiente Substâncias húmicas aquáticas: Interação com espécies metálicas 2003 1 1 São Paulo: Editora Unesp 37 39

